# Abdominal Dropsy in a Young Subject

**Published:** 1889-12

**Authors:** James Wright Putnam

**Affiliations:** Buffalo, N. Y.


### ABDOMINAL DROPSY IN A YOUNG SUBJECT.

By JAMES WRIGHT PUTNAM, M. D., Buffalo, N. Y.

SOME time ago, I was invited by Dr. Burwell, of this city, to see the following case:

A boy, eleven months old, with acute general dropsy. The scalp, hands, arms, legs, and ankles all pitted on pressure. The abdomen was greatly distended with fluid. There was no history of previous ill-health. The bowels moved naturally every day. There was no suppression of urine. There had been no cutaneous trouble.

Small powders of jalap and cream of tartar were prescribed. These operated freely, and reduced the edema of the extremities to a marked extent. The ascites, however, was undiminished, and we accordingly decided to tap.

We drew off one pint of fluid, which was straw-colored, and did not differ from the usual ascitic fluid. The child made a complete recovery.

I report this case, as I believe it unusual to find dropsy in children under a year old.

A HYPNOTIC SOCIETY.—A number of London physicians have organized a Hypnotic Society, for the purpose of studying hypnotic phenomena, and securing a law prohibiting public séances of hypnotism, mesmerism, and the like.—*Phila. Med. and Surg. Reporter.*

